# Barriers and future improvements of workplace-based learning in Korean medicine clinical clerkship: perspectives of graduates

**DOI:** 10.1186/s12909-024-05288-3

**Published:** 2024-05-23

**Authors:** Eunbyul Cho, Do-Eun Lee, Dongha Lee, Hyun-Jong Jung

**Affiliations:** 1 KM Science Research Division, Korea Institute of Oriental Medicine, Yuseong-daero, Yuseong-gu, Daejeon, 1672 Republic of Korea; 2 Department of Neuropsychiatry Medicine, College of Korean Medicine, Wonkwang University, Iksan, Republic of Korea; 3 College of Korean Medicine, Wonkwang University, Iksan, Republic of Korea; 4 Department of Diagnostics, College of Korean Medicine, Wonkwang University, Iksan, Republic of Korea

**Keywords:** Clinical clerkship, Competency, Education, Traditional Korean Medicine, Workplace-based learning

## Abstract

**Background:**

Workplace-based learning (WPBL) has emerged as an essential practice in healthcare education. However, WPBL is rarely implemented in Korean medicine (KM) due to the passive attitude of teachers and possible violation of medical laws that limit the participation of trainees in medical treatment. In this study, we implemented WPBL in the clinical clerkship of Acupuncture and Moxibustion Medicine at a single College of KM and explored the barriers and future improvements of WPBL.

**Methods:**

The WPBL was implemented from January to July 2019. During the clerkship, each senior student was assigned an inpatient at the university hospital. WPBL was conducted as follows: patient presentation by the supervisor, interaction with the patient at the bedside, preparation of medical records, oral case presentation, and discussion with feedback. The student performed a physical examination and review of systems as a clinical task. In addition, six doctors of KM who are currently practicing after three years of WPBL were interviewed in September 2022 to investigate the real-world effects and unmet needs of WPBL in their workplaces.

**Results:**

Two major themes identified from the interview were: “the experience of novice doctors of KM with KM practice” and “Current state of KM clinical education.” The five subcategories were: “Clinical competency priorities vary according to the KM workplace,” “Difficulties faced by doctors of KM immediately after graduation,” “WPBL experience of the interviewees,” “Necessary but difficult to implement real patient learning,” and “Unmet needs for clinical clerkship in KM.”

**Conclusion:**

It is essential to consider the unique characteristics of KM practice and the duties required in various workplaces for successful WPBL. We anticipate our study to be a starting point for improving the WPBL and addressing the unmet needs in KM clinical education.

**Supplementary Information:**

The online version contains supplementary material available at 10.1186/s12909-024-05288-3.

## Background

Before the twentieth century, medical and Korean medicine (KM) education was mainly based on experience through apprenticeship [1, 2]. Following the publication of the Flexner Report, medical education has shifted to a lecture-oriented form based on scientific theories and principles [2, 3]. However, the need for workplace-based learning (WPBL) has arisen from concerns regarding the increasing disconnect between medical education and clinical practice [4]. WPBL refers to learning while performing practical tasks in the field [4, 5]. WPBL enables trainees to develop skills pertaining to clinical tasks, communication, clinical reasoning, patient management, professionalism, and attitudes toward inpatients [6]. Direct observation by teachers in the workplace is an important assessment tool in competency-based education and is necessary for deliberate practice [7].

In South Korea, with the dual medical system, doctors of KM not only use the International Classification of Diseases (ICD) to diagnose like conventional medicine doctors, but also use the unique pattern recognition and systematic review of the body systems for diagnosis [8]. Doctors of KM exclusively use traditional treatments such as acupuncture and herbal medicine, and most of their services are reimbursed by the national health insurance [9]. KM education has gradually become competency-based since the publication of the secondary job analysis and competency modeling for doctor of KM [10, 11]. Studies on the objective structured clinical examination (OSCE) [12–15] and the clinical performance examination (CPX) [16–18] for clinical practice education have been reported as Colleges of KM are required to use these assessment tools for clinical training [19, 20]. However, a major limitation of using standardised patients is that their conditions are “simulated” according to a scenario. Moreover, they cannot represent pathological physical signs, are expensive, and require recruitment and training [17, 21]. Therefore, the active participation of the trainees in clinical practice is encouraged by the new accreditation standards for Colleges of KM [22].

According to the Korean medical law, students in South Korea can conduct medical treatment related to their field of study under the guidance and supervision of an academic advisor. However, active participation of students in medical care during clinical clerkship has been reported to be limited due to the patients’ negative perceptions of students practicing in a clerkship setting; in addition, students cannot be paid for their medical practice [23]. Patient safety issues [24] and limited face-to-face contact with patients since COVID-19 [25] are also factors limiting experience with real patients. Due to these barriers, KM clinical clerkship is implemented passively at present, such as observing patient care or preparation of medical records [26]. However, future doctors of KM need sufficient practice with real patients before licensing, as only about 20% of graduates later become specialists through hospital training each year, and the vast majority are employed in primary healthcare institutions (PHIs) immediately after graduation [27, 28]. However, studies have yet to be reported on the feasible methods and effects of implementing WPBL during the clinical clerkship of KM. We aimed to propose the implementation of WPBL at a single University KM hospital and explore the barriers to the implementation of WPBL and future improvements required in KM clinical clerkship by interviewing alumni.

## Methods

This study was conducted as a qualitative case study. We used a qualitative thematic analysis method to obtain an in-depth understanding of the students’ experiences with WPBL in KM clinical clerkship and identify future improvements. Thematic analysis, a foundation of qualitative research methods, is an approach to identifying and analysing recurring patterns in qualitative data without being limited to a specific paradigmatic orientation [29].

### Development and implementation of WPBL

#### Design of WPBL

The learning objectives of WPBL were designed by one author (EC), who was a resident of the Department of Acupuncture and Moxibustion Medicine, the primary care physician for inpatients, and the supervisor of this WPBL, based on the competency modeling for doctor of KM [11] and were reviewed by the head professor of the department. Then, training contents were designed to accomplish the learning objectives. Patient presentation by the supervisor, interaction with a real patient at the bedside, case presentation, and writing medical records were set as four components of WPBL (Table 1). The Department of Acupuncture and Moxibustion Medicine has a special focus on patients with musculoskeletal or neurological disorders. We chose the department as the research field because musculoskeletal disorders, which are the department’s primary focus, account for the largest portion (74.8% in 2022 in South Korea) of KM treatment [30]. We aimed to provide adequate training for treating musculoskeletal disorders for future doctors of KM.

**Table 1:** Learning objectives for the workplace-based learning and the related competencies from the 2016 Competency Modeling for doctor of Korean medicine

Training contents	Learning objectives	Competency from 2016 Competency Modeling for doctor of Korean medicine
Patient presentation by the supervisor, Interaction with a real patient at the bedside	Perform history-taking, physical examination, and review of system evaluations, select appropriate image examinations, and explain the treatment plan.	[1]-1 History taking & Physical Examination [1]-3 Integrative Care [2]-1 Physician-Patient Communication [3]-2 Emphasis on Ethical Awareness [5]-1 Patient-care Management
Case presentation	Understand and explain the inpatient’s medical history, symptoms, and physical signs.	[2]-2 Standardized Communication in between Korean Medicine Doctors
Writing medical records	Write medical records such as progress notes and medical certificates.	[1]-2 Expertise Knowledge & Practical Skills

In order to ensure that the patients do not feel overwhelmed, we assigned a single student to each patient. Patient presentation by the supervisor was to help students efficiently learn their patients’ history (Competency [1]-1 History taking) and how to manage patients’ information and records (Competency [5]-1 Patient-care Management). After patient presentation, we aimed each student to suggest one musculoskeletal or neurological examination which could be useful to identify the patient’s condition. We intended to prevent errors by making the students perform the suggested physical examination on their supervisor with immediate feedback before interacting with the patient (Competency [1]-1 Physical examination). At the bedside, to demonstrate how to take a patient’s history, we let the supervisor to first identify the primary symptom for the students. The clinical tasks assigned to each student included one physical examination, review of system, and tongue and pulse diganosis. Tongue and pulse diagnosis are important examinations that must be performed for diagnosis in KM and require extensive clinical experience to gain proficiency [31, 32]. We asked the students to diagnose the patients and develop treatment plans based on all the information gathered (Competency [1]-3 Integrative Care). The practice was also intended to let students communicate with patients and treat them with respect (Competency [2]-1 Physician-Patient Communication, [3]-2 Emphasis on Ethical Awareness). Writing medical records was included to help students organise what they had learned about the patient and document it in a fixed format. We also aimed for students to learn about the ICD codes and select an appropriate diagnosis, as doctors of KM must diagnose the patient after consultation and enter the appropriate ICD code in the chart [11], and the ICD codes are also included in the medical certificates issued by them (Competency [1]-2 Expertise Knowledge & Practical Skills). Since learning in the workplace is restricted to the clinical condition of the patient assigned to the student [4], we set a case presentation in which students described and shared their experiences to practice peer communication (Competency [2]-2 Standardized Communication in between Korean Medicine Doctors). Oral case presentation is the structured presentation of a clinical case to present important data, assessments, and plans. It is one of several methods used in workplace-based assessment [33].

#### Development of WPBL

The supervisor developed a portfolio form including the learning objectives, medical progress note, and doctor’s opinion form for the training was provided to students to record the contents of the training for a week. The learning objectives were presented in the portfolio to inform the students what they had to accomplish and promote learning [34]. The teaching materials of this WPBL were medical records, including the electronic medical records, prescriptions, and data from imaging tests.

#### Implementation

**Fig. 1:**
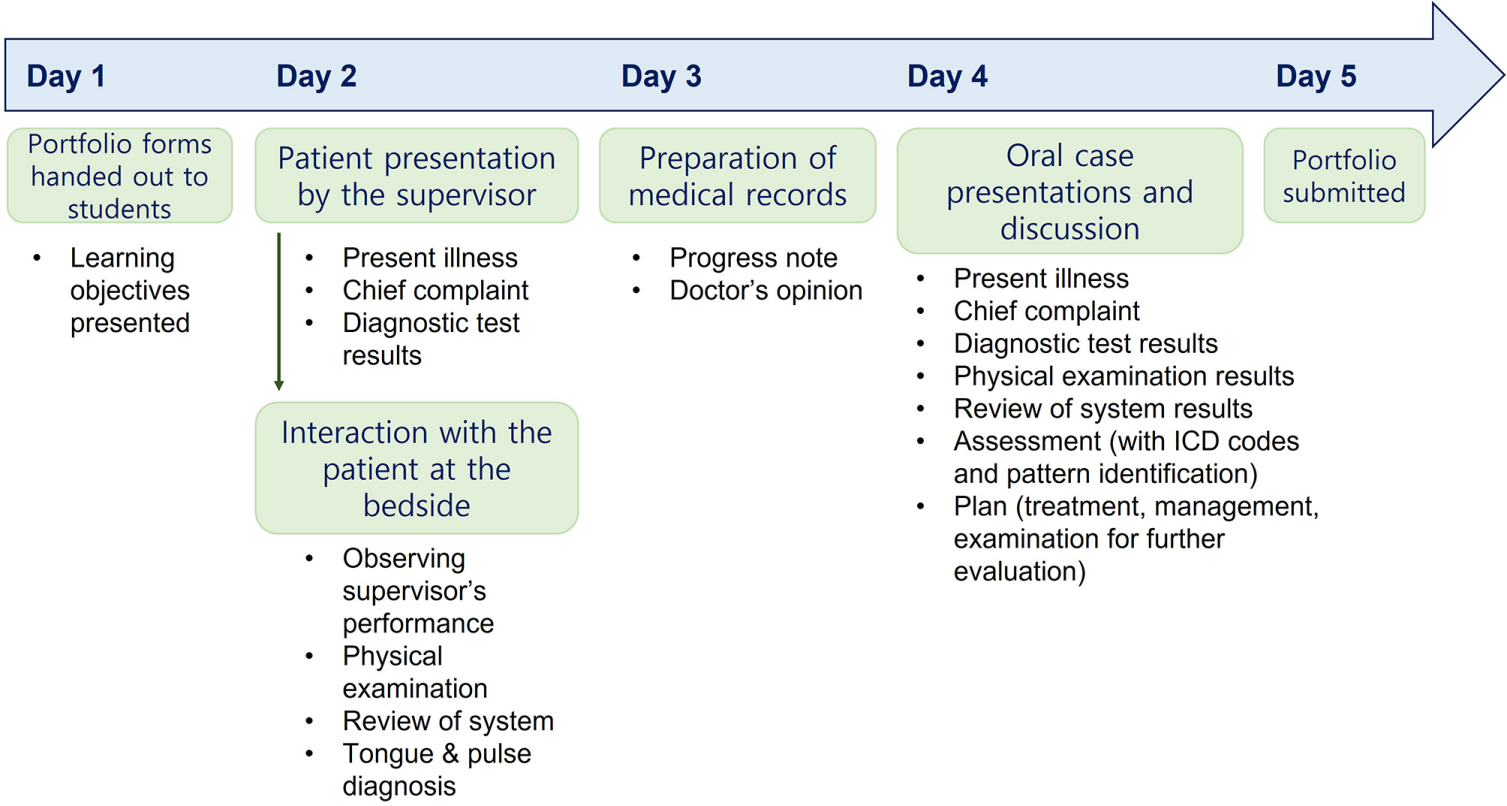
Flow chart of workplace-based learning in the present study

WPBL was conducted from January to July 2019 with the participation of 87 fourth-year students pursuing their clinical clerkship at the Wonkwang University, College of KM. Twenty-four groups comprising 3–4 students each participated in the training for five days. Each student was assigned a different patient. Inpatients who agreed to be examined by the students and were able to communicate on a daily basis participated in the WPBL. The WPBL process is presented in Fig. 1.

#### Patient presentation by the supervisor

The supervisor explained the patient’s history and chief complaints; presented diagnostic images, such as radiographs using the Picture Archiving and Communication System (PACS); explained the findings; and provided instructions regarding history-taking and the preparation of medical records. Each student was instructed to suggest one physical examination that could be performed on the patient. The suggested physical examination was subsequently performed by the patient on the supervisor before interacting with the patient to prevent medical errors. If errors were noted during the examination, immediate feedback was given to perform the examination accurately. The students were instructed to select interview contents from various Review of System (ROS) items for performing pattern identification, a diagnostic method in KM. Although the supervisor did not suggest specific ROS items, students were required to perform tongue and pulse diagnosis. The students were informed that the patient’s information should not be disclosed and that students are responsible for disclosing personal information. The supervisor’s presentation was completed in 15–20 min.

#### Bedside interactions with real patients

After the discussion, the supervisor accompanied each student to the bedside. The supervisor initially asked the patient about the chief complaint, including pain intensity, pain characteristics, and aggravating factors. Subsequently, a physical examination, ROS assessment, and tongue and pulse diagnosis were performed on the patient by the student. The students were instructed to wash their hands thoroughly before and after patient contact. All procedures at the bedside were under the supervisor’s observation and supervision. After completing the task, the student returned to the office to review their practice. The same process was repeated for the next student and designated patient. Interaction with each patient was completed in 10–15 min by each student and a total of 40–60 min for the supervisor.

#### Preparation of medical records

Each student was instructed to prepare a medical record comprising the patient’s present illness, chief complaint, results of physical examination and ROS assessment, diagnosis, and treatment plan. The next day, the students received training for preparing medical certificates and doctors’ notes. A doctor’s note form was provided to the students to write the diagnosis using the ICD 10th edition codes, the date of onset, and future treatment opinions.

#### Oral case presentations and discussion

Each student prepared and delivered an oral case presentation on the patient’s present illness from existing medical records, chief complaints as determined by observing the supervisor, results of physical examination and ROS assessment performed by the student, the diagnosis made by the student, ICD code, and pattern identification by combining all information, and the treatment plan. After each presentation, all group members and supervisors discussed the patient, and the process was completed in approximately 1 h.

#### Feedback

Explicit comments were minimised at the bedside, and simple feedback was provided in real time such that the student could revise the question or check items that were not performed. During the oral case presentation, feedback was provided primarily on the ROS, the students’ communication with the patient, the prepared medical record, and selected ICD codes.

#### Patient informed consent

We obtained informed consent from the patients to use their personal information for educational purposes. Additional verbal consent was obtained for participation in the WPBL process. None of the patients objected to or refused participation in the students’ training during the study period.

### Conduct of qualitative research

#### Study population

The graduates were set as the study population to explore the challenges faced by graduates when they started their job, whether WPBL was helpful for them in their workplace, and what aspects of WPBL need to be emphasized and improved. Additionally, the study aimed to identify the barriers to WPBL implementation and ways to improve the clinical clerkship by including university hospital residents currently responsible for clinical clerkship. In recruiting participants, purposeful sampling was applied to select study subjects from various types of workplace. This was due to the possibility of different opinions on WPBL depending on their work experience after graduation. Afterward, a snowball sampling was conducted by receiving recommendations to see if there were any doctors of KM with experience in other types of workplaces. The target of the initial purposive sampling was selected as a person who had experience as a student representative and was familiar with the work patterns of the graduates who experienced WPBL. The criteria for selecting study subjects were those who completed the WPBL and had been practicing KM for more than two years at the time of the interview.

#### Data collection

To determine whether the implementation of WPBL was beneficial to real-world clinical practice, interviews were conducted 3 years after the implementation of WPBL by a researcher (DL) with experience in qualitative studies who was not involved in the WPBL. As a doctor of KM and a specialist in KM Neuropsychiatry, the interviewer had an in-depth understanding of KM education and real-world practice. According to the purpose of the study, the researchers agreed that the main question was ‘Has WPBL helped clinical practice in the actual workplace?’ and set detailed interview question to this end. The key interview questions were: “What was the most challenging part in your workplace immediately after graduation?”, “Did WPBL help you in your clinical practice after graduation?”, “If you found WPBL helpful, what factors helped you? Do you have a specific experience that stood out to you?”, “If WPBL was not helpful, what could be improved?”, “With regard to your current workplace, what do you think you need to learn in your clerkship?”. In-depth one-on-one interviews, lasting approximately one hour per person, were conducted using an online conferencing platform and were recorded with the consent of the participants. During the interview, field notes were used to record observations, including nonverbal expressions. The WPBL supervisor (EC) transcribed the interview verbatim. The interview was continued until theoretical saturation was reached, at which point it was subjectively determined that the data collected and analysed was sufficient and no further data collection was necessary.

#### Data analysis

The interviews were analysed using a qualitative thematic analysis method. Microsoft Excel was used to analyse the transcribed raw data. There were two coders participating in concurrent data analysis. The transcribed data was reviewed repeatedly by the interviewer (DL) and subsequently summarised as meaning units. The meaning units were coded and categorised with categories and themes initially through constant comparison. The categorisation was then discussed and revised with another researcher (EC) to ensure objectivity in the analysis process and results. If opinions differed between researchers, advice was sought from an expert with experience in qualitative research unrelated to this study. Triangulation was applied to compare the field notes, transcription data, and audio recordings to ensure reliability and validity. Participants were asked to review the results obtained by the researchers through their interviews.

#### Ethical considerations

This study was reviewed and approved by the Institutional Review Board of the Wonkwang University (WKIRB-202,209-SB-070). The topic, purpose, methods of the study, and potential benefits of participating in the study were described in detail to the participants. Only doctors of KM who were willing to participate voluntarily in the study were included. The participants were informed that the results of the interviews would be published in a paper and that their responses would be anonymized. Written informed consent was obtained prior to participation. In addition, the participants were given a reward of 7$ immediately after the interview.

## Results

### General characteristics of the interview participants

The study population consisted of six participants, including five males and one female. All but one of the six participants had experience as an intern. The specialties of participants D and E are Acupuncture and Moxibustion Medicine and KM Paediatrics, respectively. At the time of the interview, three participants were public health physicians, two participants were residents, and one participant was a self-employed doctor of KM (Table 2).

**Table 2:** Sociodemographic characteristics of the participants

Participants	Age	Gender	Career (Workplace experience)
A	29	Male	Intern (KM Hospital) Public Health Physician (public health centre)*
B	29	Male	Intern (KM Hospital) Public Health Physician (public health centre)*
C	29	Male	Public Health Physician (public health centre)*
D	29	Male	Intern, Resident (KM University Hospital)*
E	32	Female	Intern, Resident (KM University Hospital)*
F	36	Male	Intern (KM Hospital) Salaried doctor of KM (KM clinic) Self-employed doctor of KM (KM clinic)*

KM, Korean medicine. *Current career and workplace.

### Perspectives from doctors of KM who have experienced WPBL

Meaningful units were extracted from the transcript. Twenty-eight codes were derived, which were categorised into five categories and two main themes (Table 3).

**Table 3:** Analysis of participants’ experience on clinical clerkship in a college of Korean Medicine

Themes	Categories	Codes
The experience of novice doctors of KM with KM practice	Clinical competency priorities vary according to the KM workplace	Patient needs and attitudes vary across healthcare organisations
		Clinical skills required in workplaces
		Detailed testing and recording required in KM hospitals
		Limitations of detailed history-taking and examination
	Difficulties faced by doctors of KM immediately after graduation	Feeling “thrown” in front of the patient
		Tasks to be completed in limited time
		Poor clinical skills
		Deciding what to do with the patient
		Low self-esteem
Current state of KM clinical education	WPBL experience of the interviewees	WPBL not memorable
		Beneficial procedure in WPBL
		Insufficient feedback
		Indirectly related to treatment
		Lack of time for self-reflection and discussion
	Necessary but difficult to implement real patient learning	The importance and benefits of interaction with real patients
		Patient discomfort with care by student
		Lack of training staff
	Unmet needs for clinical clerkship in KM	Limitations of observation
		Bridging the gap between theory and practice
		Expanding engagement in real patient care
		Practice preparation of a medical record
		Learning objectives and systemic design
		Experience with essential patient group
		Primary care-focused practice
		Implementing further CPX
		Sufficient teaching of clinical skills and OSCE
		Further practice with medical device
		Education on finance & human resources management

KM, Korean medicine; WPBL, workplace-based learning; CPX, clinical performance examination; OSCE, objective structured clinical examination.

#### Theme 1: The experience of novice doctors of KM with KM practice

##### Category 1: Clinical competency priorities vary according to the KM workplace

###### *Patient needs and attitudes vary across healthcare organisations*

Participant F, who has worked as an intern, salaried physician, and self-employed physician since graduation, reported that the needs and attitudes of patients differed according to the type of KM institutions. Patients who visit KM clinics express various needs plainly to doctors of KM; therefore, Participant F “needed a period of time to realise the adequate compromise with patients” and determine “how much to accept.”

“I had to adjust to the needs of patients presenting to KM clinic, which are different from those of patients presenting to hospitals. In hospitals…patients do not ask this and that.” (Participant F).

###### *Clinical skills required in workplaces*

The perception of the participant regarding the types of clinical skills required in KM clinics and hospitals differed. Participant F stated that therapeutic skills are required in PHIs as he had to “insert acupuncture needles quickly, accurately, and completely,” as the clinic he worked for had more than 50 patients visiting per day, and acupuncture was used as a routine treatment for all patients. Thus, proficiency in acupuncture was a mandatory requirement. In contrast, participant E, who had been an intern, struggled with being “primarily assigned to managing inpatients,” which required her to perform various clinical procedures, such as intubation, L-tube placement, and auscultation of heart sounds.

###### *Detailed testing and recording required in KM hospitals & limitations of detailed history-taking and examination*

Another difference between KM clinics and hospitals is that hospitals require more thorough testing and completeness of medical records. Since interns have a set list of things to evaluate, Participant D reported that he “noticed he forgot to check something in the list after an interview with a patient was over and returned to the office to chart.” When Participant F was an intern at a hospital, he was required to “keep a thorough record” of the patient’s presenting illness, past medical history, physical examination, and imaging findings; however, he felt that there were interviews and examinations “that were not directly related to treatment.” Since Participant F is now self-employed in a KM clinic, he is more concerned about charting “patients involved in road traffic accidents,” whose records are particularly important, and documenting that he has fulfilled his obligation to “inform” patients. He also described the difficulty of performing detailed history-taking and physical examinations for patients who were seeking an immediate cure:

“Patients with a sprained ankle, for example, seek immediate pain relief, such as an ice pack, and to be able to walk better. They did not want me to perform a detailed history-taking or physical examination…” (Participant F).

Although differential diagnosis through specific history taking and examinations is required in KM hospitals, doctors of KM employed in KM clinics face varying patient needs and are requested to provide immediate treatment effects.

##### Category 2: Difficulties faced by doctors of KM immediately after graduation

###### *Feeling “thrown” in front of the patient*

The participants reported feeling “embarrassed” and “thrown in front of patients” immediately after becoming a doctor of KM. They did not have sufficient experience with real patients during clinical clerkship or sufficient training in their workplaces after graduation.

“I did not have a lot of interaction with real patients; therefore, when I went to the clinical field and started seeing patients, I did not know what to say, what to ask. Moreover, I was not organised, so it was a bit difficult. I think I was nervous about performing clinical skills, such as acupuncture, as I did not have a lot of experience during clinical clerkship…” (Participant A).

“I was only given a quick handover for two days before I started my job at the hospital, so I did not know what test to do…” (Participant D).

Participant C, who worked as a public health physician immediately after graduation, had learned about using the “charting program” from his predecessor but had not received any training for medical treatment. Participant B stated that he would have preferred to have “a lot more practice with real patients” as he felt there was “a gap between what he learned in the college and real practice” during his first clinical experience.

###### *Tasks to be completed in limited time*

The need to complete tasks in a limited amount of time was challenging for novice doctors of KM. The participants who had previously interned in hospitals expressed this difficulty.

“During the period of transition from internship to residency, we have to prepare a lot of medical reports, including the doctor’s note in our hospital. During that period, we learn in theory, but when a patient came and I tried to prepare the medical record, it sometimes took 10–15 minutes… In the hospital, I must finish the task related to one patient quickly and move on to the next patient, but I could not do that, so I think it was very hard.” (Participant D).

“When you are actually working in a hospital, it is more important to finish the task in a limited time, so I think it was harder to prepare medical records in a short amount of time.” (Participant F).

In addition, ‘*poor clinical skills*’, since novice doctors of KM lack experience, and ‘*difficulty deciding what to do with the patient*’, especially what to ask patients and what to examine were reported as difficulties. ‘*Low self-esteem*’ as a medical professional was due to not being respected by other medical staff during internship and performing assistive tasks immediately after graduation.

#### Theme 2: Current state of KM clinical education

##### Category 1: WPBL experience of the interviewees

###### *WPBL not memorable*

Approximately three years after graduation, most of the participants could not recall the specifics of WPBL due to the passage of time; however, some participants recalled that they had interacted with real patients during the clerkship.

###### *Beneficial procedure in WPBL*

When asked to select the following four components of WPBL in order of importance: “Patient presentation by the supervisor,” “Interacting with a real patient at the bedside,” “Charting medical records,” and “Oral case presentation and discussion,” three of the current public health practitioners selected “Charting medical records” as the most important component.

Participant F, a self-employed physician, and Participant D, a resident, selected “Interacting with a real patient at the bedside” as the most important component. In contrast, Participant E, a resident, selected “Patient presentation by the supervisor” as the most important component. All participants mentioned the importance of having experience in preparing medical records, such as doctor’s notes.

“I think it is really important to have a lot of practice seeing patients and asking questions and charting, those activities have helped me treat patients in the clinic, and I wish I had more experience in charting.” (Participant A).

“When I was informed of the patient’s information by the supervisor, it was good to know what imaging tests they had undergone at other hospitals as I could get a sense of what kind of tests would be done for this condition.” (Participant E).

###### *Insufficient feedback & indirectly related to treatment*

Some participants reported that the feedback provided during WPBL was not sufficient and that “it would be nice to receive more in-depth feedback” (Participant F). Since the experiences of the participants with WPBL lacked direct relevance to the treatment, they required the supervisor’s explanations and feedback on how to use the information gathered to formulate a treatment plan.

“When you treat patients, you receive subjective and objective information and diagnose and formulate a treatment plan. Thus, just obtaining information is not the whole process…WPBL should not be limited to just taking history. Students need to understand how to use the information to treat. It is important to explain or give feedback on how to formulate a treatment plan based on the medical consultation.” (Participant F).

###### *Lack of time for self-reflection and discussion*

It was also reported that the schedule of having to interact with the patient immediately after being briefed about their condition did not give the participants sufficient time to consider how to interact with patients and that the discussion was not productive due to the different patient experiences. Since students are assigned different patients during WPBL and are not aware of the other patients’ conditions, they are less likely to understand the presentations of their peers and are less likely to ask questions.

“When I went to see the patient, I think I was pressed for time because I had to complete the assignment just after I got the patient’s information, and it would have been more memorable if I had taken more time to think about what to do with this patient.” (Participant B).

“Since the patient was known only to me and the supervisor, my colleagues could not question or agree with me…” (Participant D).

##### Category 2: Necessary but difficult to implement real patient learning

###### *The importance and benefits of interaction with real patients*

Most participants expressed the need to practice on real patients as pathological symptoms and signs can only be observed in a real patient. The experience of examining real patients and observing pathologic signs was more productive and helped the participants in their practice after graduation.

“I recall performing sensory tests with my fingers on patients with herniated intervertebral discs. Considering that I do remember what I did during WPBL, at least vaguely, I think it is important. I used the test a few times as I learned when I was an intern at the hospital…” (Participant B).

“Physical examination training, such as manual motor tests, performed by trainees on each other seemed meaningless as we only observed normal findings in each other. In contrast, during WPBL, when I observed ‘zero’ or ‘trace’ in the patients during the examination, it really stuck in my head, and it remained in my memory when I came here and completed my internal medicine turn. I performed the test on critically ill patients and was able to understand the condition and differentiate the motor grade…” (Participant D).

The participants reported that detailed examinations and records are required in KM hospitals and that tasks must be completed by them within a limited time. The participants believed that interacting with real patients during clinical clerkship gives an opportunity to practice detailed consultations and examinations, and write complete records. In addition, experience with real patients gives students an opportunity to establish their professional identity.

“You know, we did not have the opportunity to take the time to really sit down with a patient and gather the information that we were looking for, especially counsel and examine to set treatment goals and plans. If you pursue an internship at a hospital, you might have that opportunity, but if you do not, I do not think you will… I think it is really important to have the opportunity to interact with patients at the beginning so that you feel that you are doing something as a healthcare provider and you remain motivated.” (Participant F).

###### *Patient discomfort with care by student & lack of training staff*

However, the two residents, who are currently responsible for clerkship in their workplace, disagreed on whether WPBL with real patients should be mandatory, considering both the pros and cons. Participant D, a resident in the Department of Acupuncture and Moxibustion Medicine, reported that implementing WPBL is difficult when patients feel uncomfortable with students; however, Participant D also stated that “it is definitely important to interact with and do something with the patients.” In contrast, Participant E, a resident in the Department of KM paediatrics, reported that the benefits of real patient learning are not worth the risk of patient dissatisfaction, which was based largely on her experience that paediatric patients and their protectors avoided trainees. Nevertheless, both residents agreed that limited availability of staff for clinical clerkship and barriers, such as “interruptions” and “inconsistencies” in clerkship due to urgent workloads for the residents in charge of training, was a problem.

“I am a KM paediatrician, so I heard kids saying “Why is the student here, please tell her to leave,” or patients who were always willing to come in will not come in because strangers are standing in the clinic… And some parents said, “Please tell us when students are not going to be here, and we will come then,” this is how I got rejection.” (Participant E).

Participant F, who has become a self-employed physician, emphasized that while trainees may be allowed to “just observe” at his clinic, it can be detrimental to the clinic if the patients feel uncomfortable and do not return. From the perspective of a physician running a clinic, he is concerned about receiving complaints from patients and is cautious about allowing trainees to observe.

Someone is opening the curtains and looking at me? Some aged people may allow it, but in general, it is not easy to convince patients…especially when it comes to counsel with patients for prescription of herbal medicine. Since the cost of herbal medicine is expensive, I do not think it would be easy for patients to allow those strangers (students) to look at them and note their information….

##### Category 3: Unmet needs for clinical clerkship in KM

###### *Limitations of observation & bridging the gap between theory and practice*

Since “approximately 80% of current clinical practice is observation (Participant D),” just observing in the outpatient setting is “meaningless without patient information” (Participant E). Participant D was also refused the opportunity to observe by a professor who instructed him to “sit there like a sack of potatoes when there were no patients or leave when a patient came.” Thus, the participants lacked the opportunity to directly apply what they had learned in their previous five-year curriculum and observed a “gap between theory and practice.”

“I think it was very difficult for me to actually do what I had learned in theory. In terms of patient care and diagnosis, I think I have practiced a few times in some practical classes before, but I think it would be great if I experienced a lot more…” (Participant B).

###### *Expanding engagement in real patient care*

To close the gap between theory and practice, most participants suggested that students should be exposed to more practice with real patients. Further opportunities for patient consultation (Participant A) and experience of interacting with more than five real patients (Participants B and F) were suggested by the participants. Participant D, who is in charge of clinical clerkship, suggested that inpatients would most likely be targeted to increase the trainees’ learning in the workplace; however, since the opportunities may vary significantly due to different conditions of inpatients, it is important to balance practice with outpatients and inpatients.

###### *Practice preparation of a medical record*

Participant C suggested that trainees must be provided with more than two opportunities to perform the entire process of consultation and writing medical records on their own, and then receive feedback.

“Although I interned for a year, I realised that I am still not good at charting…” (Participant A).

“Just after graduation, I owe my ability to write medical records in the correct format to what the supervisor taught me during WPBL…” (Participant F).

###### *Learning objectives and systemic design*

The need to clarify the learning objectives of clinical practice in KM education and systematically design the practice curriculum was also mentioned by the participants. The participants had completed their overall clinical clerkship at three KM hospitals of a college. No learning objectives, such as knowledge, skills, and attitudes that students should be able to accomplish at the end of the training, were provided, and the content of the clerkship was organised inconsistently according to the decisions of the professors in each department. Furthermore, there was no list of core diseases, CPXs, or OSCEs that students were required to observe or perform before graduation.

“I felt the curriculum itself was very disorganised. Even if there had been some assigned tasks for each department, for instance, this department teaches A, and that department teaches B, I did not think that it was separated and organised when I was practicing…” (Participant B).

###### *Experience with essential patient group & primary care-focused practice*

Participant A proposed that undergraduates should interact with at least one patient with the primary disease of each of the eight clinical departments of KM. In contrast, Participant F, who has gained extensive experience at a local clinic, reported that although clinical training in KM curriculum focuses on inpatients in hospitals, many graduates are employed in KM clinics, and the characteristics of patients in KM clinics differ significantly from those of the patients in hospitals. Additionally, Participant C, who did not intern and only had experience working in public health, mentioned that he lacked training in identifying and referring emergency patients when performing his duties. He believed that students must be trained to determine when patients must be transferred from primary to tertiary care.

“Patients who come to KM hospitals usually bring their test results, so doctors of KM can identify at-risk patients. However, those in public health centres, who work in remote areas, cannot. I once wondered if I should transfer a patient with acute facial palsy due to the possibility of stroke. It would be better to provide more education on when to refer the patients to a higher level of care.” (Participant C).

###### *Implementing further CPX & sufficient teaching of clinical skills and OSCE*

Participant E, who had a negative attitude toward real patient learning due to patient dissatisfaction, believed that CPX is the most feasible way to improve competency level. Several CPXs are implemented with standardised patients at the hospital where Participant E is currently employed. Participant E emphasised the need to specify the required skills for each department and prepare sufficient models and infrastructure to practice various clinical skills. Clinical skill training and OSCE helped participants “apply the skills directly to clinical practice” (Participant B).

###### *Further practice with medical device & education on finance & human resources management*

The participants also emphasised the requirement for training on the use of medical devices, such as ultrasound and physical therapy devices, in clinical practice, as well as training on medical management and administration.

“I think it would be great if undergraduates could learn a little bit more broadly about the basics of tax or things about business management or human resource management, as a self-employed physician is also a person who runs a small company or a business.” (Participant F).

## Discussion

### Principal findings

WPBL enables students to experience situations they will encounter as doctors of KM in the future [4]. Without any previous reference to WPBL in KM education, we designed and implemented a step-by-step WPBL process and evaluated its effectiveness through graduate interviews. Participants employed in various types of workplaces found it significantly beneficial to receive hands-on experience with real patients before graduation. We observed that the priorities of clinical competency and WPBL components vary according to the workplace. Despite some barriers to implementation, this study suggests that the WPBL model can be improved and utilized in KM clinical clerkships, thereby developing competent future doctors of KM.

### Future improvements of the WPBL in KM education

The impact of WPBL was that students valued the experience of performing physical examinations and observing pathological signs in real patients and used them in their practice. This is particularly evident when taking into account the experiences of Participants B and D, who examined a patient with herniated intervertebral disc and a patient who had motor weakness. Most participants agreed that more opportunities are needed to interact with real patients. Nonetheless, we also identified some barriers and improvements of the WPBL model.

First of all, students need more detailed feedback and explanation regarding their performance and treatment plans. In the present study, although students were not graded on their work, we aimed to facilitate learning by observing, supervising, and providing specific and constructive feedback. However, some interviewees reported that the feedback was insufficient and that the students’ practice should be closely related to diagnosis and treatment during the discussion and feedback. The WPBL designed in this study focused on oral case presentations for just one hour on Day 4, resulting in limited time for feedback on the specifics of the treatment plans formulated by the students. It is necessary to allocate sufficient time for self-reflection and discussion, and provide high-quality supervision and feedback based on observation of students’ performance [35].

Secondly, training for the duties required in various workplaces after graudation is necessary. Our interviews showed that hospitals educate interns and residents by asking for well-structured medical records; however, this is not the case in primary care. All public health practitioners identified the preparation of medical records as the most important component of WPBL, despite the fact that two of them had internship experience. Even though meticulous charting was perceived as inconsistent with real-world practice by the participant who was self-employed in the local clinic, it is necessary for future doctors of KM to have a basic understanding of how to record their findings. In addition, one participant who did not intern, unlike other participants, mentioned that he needed more training for identifying and referring emergency patients. It is common for male doctors of KM to work as public health physicians for three years in rural underserved areas to fulfil their national defence obligations after receiving their license. Doctors of KM without internship experience may lack emergency patient experience and have difficulty performing examinations to rule out. Therefore, it is crucial to cover how to identify patients who require transfer to a higher level of care in WPBL.

### Unique characteristics of WPBL in KM education

In South Korea, most doctors of KM are employed in PHIs after graduation rather than pursuing internships or residencies. A total of 744 graduates were licensed as doctors of KM in 2020; however, only 131 who had completed internship and residency programs were qualified as specialists [36]. Instead, doctors of KM can choose among several career paths after licensing, including employed physician, self-employed in KM clinics, or practicing as interns and residents in KM hospitals [37]. Therefore, we aimed to interview participants with experiences in these various workplaces. The interview suggests that current KM clinical teaching is mainly conducted in the setting of KM hospitals. However, since most doctors of KM work in clinics, future doctors of KM must practice communicating with more patients in clinics, who “express various needs plainly”, unlike patients in KM hospitals. Furthermore, “Finance & human resources management” in the competency modeling for doctors of KM [11], which represents understanding for insurance and effective manpower management, should be covered in clinical teaching since many doctors of KM are self-employed physicians and manage their own practice [37]. We also found that while teaching clinical skills is fundamentally important, proficiency in routine treatment skills is prioritized in primary care. In contrast, a variety of skills for inpatient care is required in KM hospitals.

Clinical reasoning is often the focus of WPBL in medical education [33, 38]; however, we focused on the experience of interacting with real patients and performing physical examinations rather than clinical reasoning, as many doctors of KM encounter patients who have been examined and diagnosed in other hospitals. This is because many patients visit KM clinics as they wish to receive both Western and KM treatments [39]. Therefore, patient information, including medical history, chief complaint, and imaging test results of the patients, were presented to the students before they interacted with the patients to reflect the nature of KM practice. Instead of clinical reasoning, students were asked to conduct pattern identification, a unique diagnostic method in East Asian medicine, based on patients’ symptoms, students’ findings, and tongue and pulse diagnosis [40]. Moreover, the supervisor explained the results of the imaging and trained the students on the use of PACS. In South Korea, doctors of KM cannot order some diagnostic examinations, such as radiographs; however, they are required to have the ability to interpret the images and explain them to patients [41]. As clinical competency and simulation are currently emphasized in East Asian medicine education [20, 42], this study provides insight into how to implement WPBL in East Asian medicine, which differs from conventional medicine in terms of diagnosis and treatment.

### Strengths and limitations of this study

This is the first study to design and implement WPBL in KM education and assess the learners’ reactions through interviews. The graduates’ opinions on WPBL as well as clinical practice education and the unique characteristics of KM practice compared with those of Conventional medicine will serve as a reference for redesigning KM education to be competency-based. We also collected feedback from practitioners more than two years after graduation to assess the application of the training in the field. A limitation of this study is that the researcher who primarily conducted the interviews is a doctor of KM and the researcher who was responsible for the training participated in the study; thus, there may be experimental bias during participant recruitment, interviews, and data analysis. The responses of the participants may have been affected as they recognised that the interviewer was a doctor of KM. Although we conducted interviews until we reached theoretical saturation, we were only able to interview six participants. In addition, the lack of pre-interviews may have affected the quality of the study. Despite these limitations, this study is significant in that it provides an in-depth understanding of the impact of WPBL in real-world KM practice, the challenges faced by novice doctors of KM in clinical settings, and the unique characteristics of KM practice.

### Implications for future research and education

As this study is the beginning of the research on WPBL in KM education, various studies on WPBL with real patients are urgently required. First, the patients’ and teachers’ perceptions of WPBL must be investigated. During our interviews, we learned that some patients did not want students to be involved in their care, which leads to residents having a negative attitude toward real patient learning. It is worth exploring whether there is a difference in patients’ attitudes and needs toward doctors of KM and how they perceive students’ participation in patient care in KM clinics and hospitals. Faculty and patient encouragement are beneficial factors in improving clinical skills [43]. However, even Participant F, who reported that WPBL was helpful and is important in KM education also had a negative attitude toward having students in his own clinic due to concerns regarding patient comfort and their possible decision to not return. It is unknown whether patients who visit KM institutions will dislike the increased participation of students. Further studies are required to gain an in-depth understanding of the challenges and unmet needs of clinical teachers to promote WPBL.

Second, assessment should be conducted to determine whether the intended learning objectives are achieved by utilising WPBL in KM clinical teaching. Learners’ positive reactions to mini-clinical evaluation exercise and direct observation of procedural skills have suggested the value of workplace-based assessment as a formative performance assessment [44]. Student performance may be assessed via direct observation by the instructor, as well as a written record or oral presentation by the student [5]. Further research is required to develop specific methods and assessment tools for workplace-based assessment that can be utilised appropriately in KM settings, in light of reliability and feasibility.

Third, as reported by the participants, clear objectives and a systematic curriculum of KM clinical teaching must be formulated for competency-based learning. The participants had practiced for 24 weeks at three teaching hospitals; however, they were not adequately trained to perform the expected tasks. Although competency modeling for doctors of KM has been defined, little information is available on how to implement competency-based education in individual KM courses [45]. The patients and tasks that students should experience in the clinical clerkship should be clearly defined.

Furthermore, the lack of clinical preceptors was noted as a barrier to implementing WPBL in this interview. Skilled preceptors who can prioritize teaching and supervision are needed to provide students with sufficient opportunity to perform tasks in the clinical field. While many residents now serve as preceptors for clerkship alongside faculty, residents are also the trainees. The teaching and communication skills of preceptors required for clinical education could be developed through existing faculty development programs [46].

The significance of the study is that it provides a specific report of WPBL that can be utilised in KM clinical teaching. This learning required up to 140 min of supervisor time per week, which we believe is acceptable in light of the authentic experience gained by the students. Thus, we expect the promotion of WPBL in KM education in the future and the introduction of various workplace-based assessments.

## Conclusion

Promoting WPBL in KM education is crucial for enhancing various clinical competencies required in the KM workplaces. We implemented WPBL in the KM clinical clerkship and interviewed graduates three years later to explore the challenges they faced as novice doctors of KM and identify future improvements of WPBL. Patient discomfort with care by student and lack of training staff were identified as barriers to WPBL. Suggestions for improvement included providing sufficient feedback on student performance and discussion on synthesizing the information to set treatment plans. The findings of this study can contribute to improving the WPBL and addressing the identified unmet needs in KM clinical education.

### Electronic supplementary material

Below is the link to the electronic supplementary material.

Supplementary Material 1

Supplementary Material 2

## Data Availability

The datasets generated and analysed during the current study are available from the corresponding author upon reasonable request.

## Abbreviations

WPBL: Workplace-based learning
KM: Korean Medicine
OSCE: Objective Structured Clinical Examination
CPX: Clinical Performance Examination
PHIs: Primary healthcare institutions
ROS: Review of System
ICD: International Classification of Diseases
PACS: Picture Archiving and Communication System
