# Supplementary material for: Barriers and future improvements of workplace-based learning in Korean medicine clinical clerkship: perspectives of graduates
Source: BMC Med Educ. 2024 May 23;24:566. doi: 10.1186/s12909-024-05288-3 (PMC11119396; doi:10.1186/s12909-024-05288-3)
Supplement: Supplementary file 1 — Supplementary Material 1 [file 12909_2024_5288_MOESM1_ESM.docx]

**Appendix 1**: Physical examination items in medical progress reports in portfolios submitted by the students, which were later classified and counted by the supervisor

| Musculoskeletal examination (76) | Range of motion test (28) | Knee joint (11) |
| --- | --- | --- |
|  |  | Elbow joint (7) |
|  |  | Shoulder joint (4) |
|  |  | Ankle joint (4) |
|  |  | Hip joint (2) |
|  | Manual muscle test (20) | Lower extremity (19) |
|  |  | Upper extremity (1) |
|  | Palpation (13) | Muscle (9) |
|  |  | Skin (3) |
|  |  | Joint (1) |
|  | Others (15) | Straight leg raising test (5) |
|  |  | Percussion test (5) |
|  |  | Pinch roll test (1) |
|  |  | Hip and knee flex test (1) |
|  |  | Spurling test (1) |
|  |  | Hawkins test (1) |
|  |  | Neer test (1) |
| Neurologic examination (6) | Deep tendon reflex (3) | |
|  | Tinel sign (2) | |
|  | Pupil reflex (1) | |

Numbers in parentheses indicate frequency.
