# Supplementary material for: Barriers and future improvements of workplace-based learning in Korean medicine clinical clerkship: perspectives of graduates
Source: BMC Med Educ. 2024 May 23;24:566. doi: 10.1186/s12909-024-05288-3 (PMC11119396; doi:10.1186/s12909-024-05288-3)
Supplement: Supplementary file 2 — Supplementary Material 2 [file 12909_2024_5288_MOESM2_ESM.docx]

**Appendix 2**: Items checked recorded in portfolios by students while interacting with patients, which were later identified, classified and counted according to the review of system (ROS) framework* and diagnostic criteria for pattern identification* by the supervisor

| Items of ROS | Frequency and percentage of each item in the total |  | Items for pattern identification | Frequency and percentage of each item in the total |
| --- | --- | --- | --- | --- |
| Gastrointestinal | 197 (46.7%) |  | Defecation | 74 (17.5%) |
| Urinary | 70 (16.6%) |  | Urination | 70 (16.6%) |
| Neuropsychiatry | 65 (15.4%) |  | Appetite | 69 (16.4%) |
| General | 54 (12.8%) |  | Sleep | 64 (15.2%) |
| HEENT | 26 (6.2%) |  | Digestion | 50 (11.8%) |
| Respiratory | 6 (1.4%) |  | Cold/Heat | 34 (8.1%) |
| Cardiac/Chest | 2 (0.5%) |  | Sweats | 12 (2.8%) |
| Skin | 2 (0.5%) |  | Face | 11 (2.6%) |
| Total | 422 |  | Head | 10 (2.4%) |
|  |  |  | Cough/Sputum | 6 (1.4%) |
|  |  |  | Others | 6 (1.4%) |
|  |  |  | Abdomen | 4 (0.9%) |
|  |  |  | Mental/Psychological | 3 (0.7%) |
|  |  |  | Eye | 2 (0.5%) |
|  |  |  | Chest | 2 (0.5%) |
|  |  |  | Speech | 1 (0.2%) |
|  |  |  | Nose | 1 (0.2%) |
|  |  |  | Throat | 1 (0.2%) |
|  |  |  | Nails | 1 (0.2%) |
|  |  |  | Skin | 1 (0.2%) |
|  |  |  | Total | 422 |

ROS, review of system; HEENT, Head, eyes, ears, nose and throat.

*The following literature was referenced: NEO Handbook Editorial Board. Neo intern handbook. Paju(Korea): Koonja publishing company; 2016.
